# Comparative Effectiveness of Chemotherapy in Elderly Patients with Metastatic Colorectal Cancer

**DOI:** 10.1007/s12029-012-9450-x

**Published:** 2012-11-07

**Authors:** Sacha Satram-Hoang, Luen Lee, Shui Yu, Sridhar R. Guduru, Ashokvardhan R. Gunuganti, Carolina Reyes, Edward McKenna

**Affiliations:** 1 Q.D. Research, Inc, 8789 Auburn Folsom Road C501, Granite Bay, 95746 CA USA; 2 Genentech, Inc, South San Francisco, CA USA

**Keywords:** Metastatic colorectal cancer, Chemotherapy, Elderly patients, Survival, Treatment-related complications

## Abstract

**Purpose:**

Treatment advances have improved outcomes in clinical trials of patients with metastatic colorectal cancer (mCRC). Less is known about these effects for patients in real-world settings. This study evaluated treatment patterns and survival in older, demographically diverse patients with mCRC.

**Methods:**

A retrospective cohort analysis was performed for 4,250 patients from January 1, 2000 to December 31, 2007 using linked Surveillance, Epidemiology, and End Results-Medicare database. Patients were ≥66 years, enrolled in Medicare parts A and B, and received first-line treatment with fluorouracil and leucovorin (5-FU/LV), capecitabine (CAP), 5-FU/LV plus oxaliplatin (FOLFOX), or CAP and oxaliplatin (CAPOX). Cox regression with backward elimination and propensity score-weighted Cox regression estimated relative risk of death. Date of last follow-up was December 2009. Statistical comparisons were made between 5-FU/LV vs. CAP and FOLFOX vs. CAPOX.

**Results:**

Compared to 5-FU/LV, patients treated with CAP were older (mean age 78 vs. 76; *P* < 0.0001) and more likely female (61 vs. 54 %; *P* = 0.0017), while patients receiving CAPOX and FOLFOX were similar in age (mean age 74 vs. 73; *P* = 0.0924). Complications requiring medical resource utilization following initiation of therapy were significantly higher among patients administered with 5-FU/LV (54 %) vs. CAP (17 %; *P* < 0.0001) and FOLFOX (75 %) vs. CAPOX (57 %; *P* < 0.0001). The multivariate analysis revealed no significant differences in survival between 5-FU/LV and CAP and between FOLFOX and CAPOX.

**Conclusions:**

Overall survival was comparable between CAP and 5-FU/LV and between CAPOX and FOLFOX with fewer complications requiring medical resource utilization associated with CAP and CAPOX, thus confirming clinical trial results.

**Electronic supplementary material:**

The online version of this article (doi:10.1007/s12029-012-9450-x) contains supplementary material, which is available to authorized users.

## Introduction

Colorectal cancer (CRC) is a disease of the elderly with a median age at diagnosis of 70 years and median age at death of 75 years [1]. It is the third most frequently diagnosed cancer as well as the third leading cause of cancer mortality in men and women in the USA [2]. Approximately 20 % of patients are diagnosed with metastatic disease with 5-year survival rates of 11.7 % [1].

For the past 50 years, the mainstay of systemic treatment for advanced or metastatic CRC (mCRC) has been fluoropyrimidines (FP) administered as monotherapy or in combination with leucovorin (LV) or newer agents such as irinotecan (IFL) and oxaliplatin [3, 4]. Clinical trials and meta-analyses demonstrate that 5-fluorouracil (5-FU) and LV (5-FU/LV) improve response rates (RRs) and survival among patients with mCRC [3, 5]. Randomized controlled trials have also established the efficacy of 5-FU/LV plus oxaliplatin (FOLFOX) with significant improvements in RR and progression-free survival (PFS) when administered as first-line therapy for patients with advanced CRC [6].

Capecitabine (CAP) is an oral fluoropyrimidine that is converted to 5-FU. Two randomized, non-blinded phase 3 trials compared single-agent CAP with 5-FU/LV as first-line therapy of patients with mCRC and established that CAP was at least as active as 5-FU/LV in achieving an objective tumor RR, [7, 8] and PFS and overall survival (OS) were equivalent between treatment arms in a prospective pooled analysis of two similarly designed phase 3 trials [9]. Capecitabine and oxaliplatin (CAPOX) have demonstrated clinical activity in multiple clinical trials as first-line treatment for patients with mCRC [10–17] and provide comparable clinical outcomes to FOLFOX [12, 18] and infusional 5-FU/oxaliplatin [19].

Historically, elderly patients have been underrepresented in clinical trials with only one quarter to one third of potentially eligible older patients enrolled in cancer clinical trials [20–22]. This presents a significant challenge to efforts to evaluate treatment efficacy and safety in elderly patients [22]. Furthermore, there is limited knowledge about the use of recommended newer agents for the treatment of mCRC in community settings, particularly for older and demographically diverse patient populations [23]. However, there is evidence to suggest variations in the management of patients with all stages of CRC with several studies reporting lower rates of chemotherapy for older patients [23–27] and almost 30 % of stage III and IV patients were less likely to receive guideline-recommended therapies [27]. An analysis of patients in the National Cancer Data Base who were treated for CRC from 2003 to 2007 revealed that 25.9 % of patients with stage IV disease received no chemotherapy and older patients with preexisting comorbid conditions were at increased risk of under-treatment [23]. Comorbid health conditions and older age appear to influence physicians' choice of treatment regimen for all stages of CRC with older patients more likely to receive shorter chemotherapy regimens with less toxicity [23–27]. The goal of this study was to evaluate treatment patterns, OS, and frequency of complications requiring medical resource utilization in older, demographically diverse patients undergoing treatment for mCRC.

## Methods

### Data Sources

We utilized population-based claims data from the Surveillance, Epidemiology, and End Results (SEER)–Medicare linked database. The SEER–Medicare database is a collaborative effort of the National Cancer Institute, the SEER registries, and the Centers for Medicare & Medicaid Services. As detailed elsewhere [28], this database provides information on Medicare patients included in SEER, a collection of 18 population-based cancer registries of incident cases from diverse geographic areas representative of approximately 28 % of the US population. All incident cancer patients reported to SEER registries are cross-matched with a master file of Medicare enrollment [29]. Approximately 97 % of persons 65 years or older are eligible for Medicare with all beneficiaries eligible for part A coverage including inpatient care, skilled nursing, home healthcare, and hospice care. Approximately 95 % of beneficiaries also subscribe to part B, which covers physician services and outpatient care. The SEER–Medicare linkage includes all Medicare-eligible persons in the SEER database through 2007 and their Medicare claims for part A (inpatient care) and part B (outpatient and physician services) through 2009. Institutional review board approval for this study was waived because the SEER–Medicare database does not include personal identifiers.

### Study Population

Eligibility criteria for study inclusion included: (1) a first primary diagnosis of stage IIIB, IIIC, or IV CRC from January 1, 2000 through December 31, 2007, (2) age ≥66 years, (3) treatment with any oral or infused chemotherapy after diagnosis, and (4) survival time ≥60 days following the date of first-line chemotherapy initiation. We eliminated patients whose survival was less than 60 days to minimize the introduction of immortal time bias into the analyses [30]. Patients were also excluded if their date of death was recorded prior to or in the same month as diagnosis, enrollment in Medicare parts A and B for less than 12 months before the diagnosis date, enrollment in a health maintenance organization (HMO) for any period of the 12 months prior to diagnosis (because data were unavailable for this time), two or more claims for chemotherapy prior to diagnosis (to ensure that the cases were previously untreated), and finally, cases were excluded if they underwent primary resection of the tumor prior to initiating chemotherapy (to eliminate potential adjuvant cases). See Supplementary Fig. 1 for schematic of inclusion/exclusion process.

### Study Variables

The SEER program routinely collects data on patient demographics including age, race/ethnicity, residence, and socioeconomic status (income and education per census tract), primary tumor site, tumor morphology, stage at diagnosis, first course of treatment, and follow-up for vital status. Median annual household income at the census tract level and percentage of the adult population who completed specific levels of education at the zip code level were used as a proxy for socioeconomic status. SEER site codes identified colon and rectum cancer cases. The American Joint Committee on Cancer and SEER stage groupings were used to identify stage at diagnosis.

To identify claims for chemotherapy administration, [31] data were abstracted from four merged SEER–Medicare claims files including (1) Medicare provider analysis and review, (2) carrier claims from the National Claims History, (3) outpatient claims (OUTSAF), and (4) durable medical equipment (DME). Claims for oral equivalents of intravenous chemotherapies (i.e., capecitabine) were identified in the DME file. Chemotherapy agents were characterized and quantified using International Classification of Disease (ICD) diagnosis codes, ICD procedural codes, Current Procedural Terminology codes, Healthcare Common Procedural Coding System (HCPCS) codes, and revenue center codes. Chemotherapy claims were searched for specific drug codes to identify the type of chemotherapy used. The absence of these claims indicated lack of treatment. The first chemotherapy claim following diagnosis indicated the start of therapy. Patients were classified into one of four treatment groups (5-FU/LV, CAP, FOLFOX, and CAPOX) based on all chemotherapy administered during the first 60 days after treatment initiation.

Medicare claims identified patients who underwent primary resection of the tumor prior to initiating chemotherapy. Surgical procedures included hemicolectomy, subtotal colectomy, and total colectomy. Claims filed 1 year before diagnosis were used to determine baseline comorbidity burden. Comorbidities were aggregated to formulate the National Cancer Institute (NCI) comorbidity index, a revised version of the Charlson comorbidity index [32]. The incidence of specific treatment-related complications (anemia, neutropenia, nausea/vomiting, diarrhea, and dehydration) requiring medical resource utilization was assessed 180 days following treatment initiation. This 180-day period was selected as appropriate based on the National Comprehensive Cancer Network guidelines that recommend 6 months of adjuvant treatment for stage II and III CRC or for stage IV stable disease [33]. Anemia was defined by the condition-specific ICD-9 diagnosis codes, a revenue center code or HCPCS code for a red blood cell transfusion, or a revenue center code or J-code for an erythropoiesis-stimulating agent. Other treatment complications were defined using the condition-specific ICD-9 codes in both inpatient and outpatient Medicare claims records (codes available upon request).

The date of death was determined by using the Medicare date or the SEER date of death if the Medicare date was missing. All other patients were assumed to be alive at the end of the follow-up period on December 31, 2009, although they may have been censored earlier for other reasons such as development of a second primary cancer or Medicare claims no longer available.

### Statistical Analysis

All statistical analyses were performed using SAS software, version 9.1.3 (SAS Institute Inc., Cary, NC). Statistical comparisons were made between 5-FU/LV vs. CAP and FOLFOX vs. CAPOX. Descriptive statistics were calculated for demographic and clinical variables and treatment patterns. Differences between treatment groups were evaluated with chi-square tests for categorical variables and analysis of variance or *t* test for continuous variables. A *P* value <0.05 was considered statistically significant.

In the survival analysis to assess overall risk of death, we compared two approaches as a sensitivity exercise: (1) multivariate Cox proportional hazards regression and (2) propensity score-weighted Cox proportional hazards regression. In the first model, we adjusted for confounders that were selected from demographic and clinical characteristics using the backward elimination strategy [34]. In the second model, multinomial logistic regression was used to calculate a propensity score for each individual. The propensity score is the conditional probability of each patient receiving a specific treatment based on baseline characteristics [35]. The effect of the propensity score weights was to balance the groups to reduce potential bias associated with treatment selection. A propensity score-weighted Cox proportional hazards regression model was fitted to compare overall survival between treatment groups. Follow-up was calculated beginning on the date of treatment initiation up until the first occurrence of a censoring event: date of death, development of a second primary tumor, last date for which Medicare claims were available, or last date of the follow-up period (December 31, 2009).

## Results

### Demographic and Clinical Characteristics

Of the 7,061 patients who met all study inclusion criteria, 2,213 were treated with 5-FU/LV, 1,298 received FOLFOX, 617 were administered with CAP, and 122 received CAPOX (Table 1). Of the remaining 2,811 patients, about 28 % received irinotecan-based therapy, 57 % received other types of chemotherapy, and 15 % received an unknown type of chemotherapy. Compared with patients administered with 5-FU/LV, patients treated with CAP were older, more likely female, diagnosed with stage IIIB/C disease, and had higher tumor grade. A higher proportion of patients treated with CAPOX were older and had stage IV disease compared with those treated with FOLFOX. Higher rates of treatment with CAP and CAPOX were evident for patients residing in the west and those with higher levels of income and education compared with patients administered with 5-FU/LV and FOLFOX, respectively.

**Table 1:** Demographic and clinical characteristics at baseline

Characteristic	CAP (*N* = 617)		5-FU/LV (*N* = 2,213)		*P* value	CAPOX (*N* = 122)		FOLFOX (*N* = 1,298)		*P* value
	*n*	%	*n*	%		*n*	%	*n*	%	
Age at diagnosis										
66–70	96	15.6	503	22.7	<0.0001	43	35.2	495	38.1	0.0924
71–75	137	22.2	641	29.0		30	24.6	419	32.3	
76–80	160	25.9	591	26.7		38	31.1	296	22.8	
>80	224	36.3	478	21.6		11	9.0	88	6.8	
Sex										
Male	243	39.4	1,029	46.5	0.0017	56	45.9	632	48.7	0.5557
Female	374	60.6	1,184	53.5		66	54.1	666	51.3	
Race/ethnicity										
White	499	80.9	1,850	83.6	0.1115	104	85.2	1,130	87.1	0.5708
Non-White	118	19.1	363	16.4		18	14.8	168	12.9	
Stage at diagnosis										
Stage IIIB/C	381	61.8	1,027	46.4	<0.0001	79	64.8	962	74.1	0.0254
Stage IV	236	38.2	1,186	53.6		43	35.2	336	25.9	
Tumor grade										
Grade 1	33	5.3	96	4.3	0.0145	81^a^	66.4	69	5.3	0.9117
Grade 2	351	56.9	1,397	63.1				790	60.9	
Grade 3	189	30.6	617	27.9		41^a^	33.6	373	28.7	
Grade 4	18	2.9	33	1.5				27	2.1	
Unknown	26	4.2	70	3.2				39	3.0	
Comorbidity score										
0	338	54.8	1,307	59.1	0.1999	78	63.9	806	62.1	0.9469
1	173	28.0	562	25.4		32	26.2	342	26.3	
2	60	9.7	213	9.6		12^a^	9.9	97	7.5	
≥3	46	7.5	131	5.9				53	4.1	
Geographic region										
Midwest	81	13.1	261	11.8	0.0001	17^a^	13.9	170	13.1	<0.0001
Northeast	34	5.5	145	6.6				85	6.5	
South	251	40.7	1,102	49.8		30	24.6	567	43.7	
West	251	40.7	705	31.9		75	61.5	476	36.7	
Median income quartiles										
1—low	130	21.1	576	26.0	0.0002	21	17.2	334	25.7	0.1633
2	145	23.5	559	25.3		35	28.7	319	24.6	
3	146	23.7	557	25.2		30	24.6	325	25.0	
4—high	195	31.6	509	23.0		36	29.5	318	24.5	
Education	Mean	95 % CI	Mean	95 % CI	*P* value	Mean	95 % CI	Mean	95 % CI	*P* value
Less than high school, %	18.7	[17.73, 19.76]	20.0	[19.46, 20.46]	0.0298	17.1	[14.84, 19.41]	18.5	[17.92, 19.17]	0.1966
High school only, %	26.7	[25.89, 27.43]	28.7	[28.33, 29.10]	<0.0001	23.1	[21.36, 24.88]	28.1	[27.54, 28.57]	<0.0001
Some college, %	27.5	[26.91, 28.00]	27.1	[26.87, 27.43]	0.3359	28.6	[27.25, 29.91]	28.0	[27.66, 28.41]	0.4058
At least a college degree, %	27.1	[25.81, 28.47]	24.2	[23.55, 24.81]	<0.0001	31.2	[27.99, 34.36]	25.4	[24.50, 26.24]	0.0002

^a^Cells with counts of less than 11 are combined in compliance with the National Cancer Institute data use agreement for small cell sizes

### Treatment Patterns

**Fig 1:**
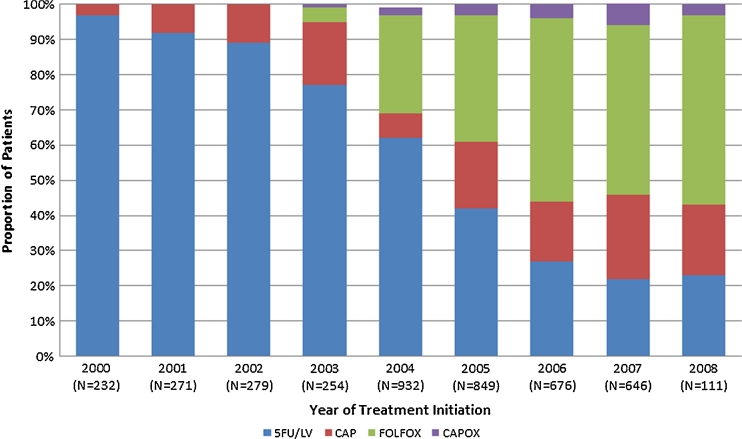
Type of treatment by year of initiation

Use of CAP, CAPOX, and FOLFOX increased over time while treatment with 5-FU/LV decreased during the same time period (Fig. 1). The mean time to initiation of chemotherapy following diagnosis was similar between treatment groups ranging from 74 days for 5-FU/LV, 77 days for CAPOX, 78 days for FOLFOX, and 81 days for CAP (Table 2). The mean duration of treatment was longer for those administered with 5-FU/LV (147 days) compared with 128 days for the CAP group (*P* < 0.0001) while there was no significant difference in duration of treatment with CAPOX (143 days) and FOLFOX (151 days; *P* = 0.2335).

**Table 2:** Time to first-line treatment and duration of first-line treatment

	*N*	Mean	SD	Median	Min	Max	*P* value
Time to treatment^a^, days							
CAP	545	80.68	34.93	74	7	179	<0.0001
5-FU/LV	2,117	73.80	29.63	69	1	177	
CAPOX	120	77.11	33.68	72	15	175	0.8811
FOLFOX	1,255	77.53	29.18	72	14	175	
Duration of treatment^b^, days							
CAP	603	128.41	76.91	118	30	357	<0.0001
5-FU/LV	2,140	147.48	75.01	143	30	365	
CAPOX	119	143.43	64.45	141	31	300	0.2335
FOLFOX	1,285	150.93	65.78	157	30	360	

^a^Time to treatment initiation defined as “time from diagnosis” to “date of first chemotherapy claim”

^b^Duration of treatment defined as time from date of first chemotherapy claim to 30 days following last administration of first-line agent, or to the day prior to second-line treatment initiation or 30 days following last administration of first-line agent if gap in therapy is >90 days

### Complications Requiring Medical Resource Utilization

The overall rate of complications requiring medical resource utilization (Table 3) within 180 days after treatment initiation was higher for patients treated with 5-FU/LV (54.3 %) and FOLFOX (74.9 %) compared with CAP (17.2 %) and CAPOX (56.6 %), respectively (<0.0001 for both comparisons). The three most frequent complications requiring medical resource utilization were anemia, nausea/vomiting, and diarrhea with significantly higher rates for 5-FU/LV vs. CAP and FOLFOX vs. CAPOX (*P* < 0.0001 for all comparisons).

**Table 3:** Incidence of medical resource utilization related treatment complications requiring intervention (hospitalization or treatment) within 180 days after initiation of treatment

Adverse events	CAP (*N* = 617)		5-FU/LV (*N* = 2,213)		*P* value	CAPOX (*N* = 122)		FOLFOX (*N* = 1,298)		*P* value
	*n*	%	*n*	%		*n*	%	*n*	%	
Any treatment-related complications	106	17.2	1,201	54.3	<0.0001	69	56.6	972	74.9	<0.0001
Anemia	80	13.0	879	39.7	<0.0001	38	31.1	699	53.9	<0.0001
Nausea/vomiting	24	3.9	453	20.5	<0.0001	36	29.5	494	38.1	<0.0001
Diarrhea	18	2.9	157	7.1	<0.0001	−^a^	−^a^	89	6.9	<0.0001
Neutropenia	−^a^	−^a^	13	0.6	0.0013	−^a^	−^a^	121	9.3	<0.0001
Dehydration	−^a^	−^a^	52	2.3	<0.0001	−^a^	−^a^	108	8.3	<0.0001

^a^Cells with counts of less than 11 are suppressed in compliance with the National Cancer Institute's data use agreement for small cell sizes

### Survival Outcomes

**Fig 2:**
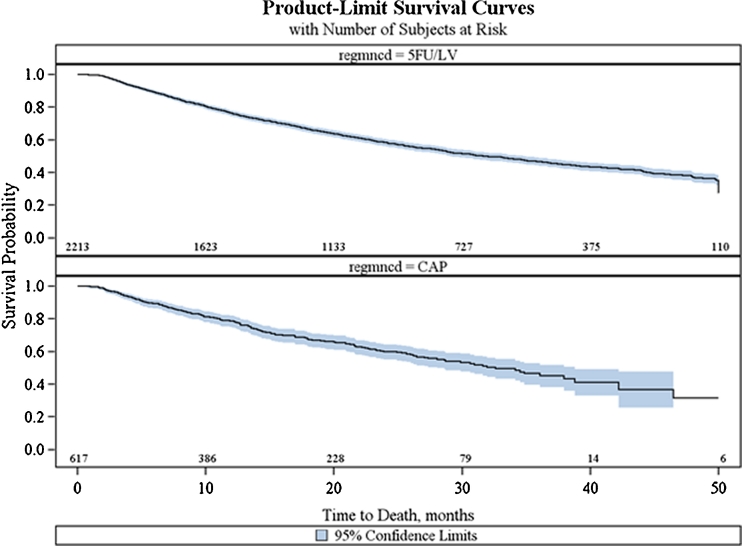
Kaplan–Meier curve of overall survival by treatment (CAP vs. 5-FU/LV)

The median survival time was 32.6 months (95 % CI, 28.1–38.8) in the CAP group and 31.9 months (95 % CI, 29.1–34.9) in the 5-FU/LV group (log rank *P* = 0.6683; Fig. 2). The multivariate Cox regression survival analysis (Table 4) revealed no significant differences in risk of death between CAP compared with 5-FU/LV (HR, 0.919; 95 % CI, 0.799–1.058; *P* = 0.2396). This finding was confirmed in the propensity-weighted Cox regression. The full Cox model included treatment, age, sex, race, positive lymph nodes, tumor grade, comorbidity score, geographic region, and income. After backward elimination, age, greater number of positive lymph nodes, higher tumor grade, and lower income levels were identified as significant predictors of mortality.

**Table 4:** Multivariate Cox regression of overall survival (CAP vs. 5-FU/LV)

Covariates	*N*	Multivariate Cox regression reduced model^a^			Propensity weighted Cox regression^b^		
		HR	95 % CI	*P* value	HR	95 % CI	*P* value
Treatment							
5-FU/LV (ref)	2,213	1.000			1.000		
CAP	617	0.919	0.799–1.058	0.2396	0.868	0.753–0.999	0.0487
Age at diagnosis							
66–70 (ref)	599	1.000					
71–75	778	1.078	0.930–1.250	0.3184			
76–80	751	1.310	1.131–1.519	0.0003			
> 80	702	1.455	1.250–1.694	<0.0001			
Positive lymph nodes							
0 (ref)	278	1.000					
1–3	1,362	0.710	0.599–0.841	<0.0001			
≥4	1,024	1.275	1.077–1.508	0.0048			
Tumor grade							
1–3 (ref)	1,877	1.000					
3–4	857	1.253	1.123–1.398	<0.0001			
Median income quartiles							
1 (low) (ref)	768	1.000					
2	720	0.949	0.825–1.092	0.4668			
3	656	0.858	0.742–0.993	0.0402			
4 (high)	673	0.909	0.787–1.049	0.1927			

^a^Reduced model by backward elimination. Full model included age, sex, race, positive lymph nodes, tumor grade, comorbidity score, geographic region, and income

^b^Propensity score weighted for age, sex, race, positive lymph nodes, tumor grade, comorbidity score, geographic region, and income

**Fig 3:**
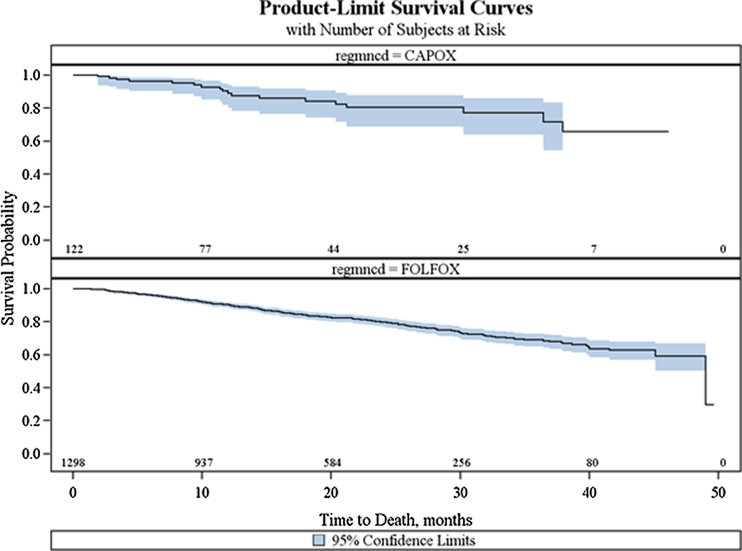
Kaplan–Meier curve of overall survival by treatment (CAPOX vs. FOLFOX)

Figure 3 demonstrates that while the median survival time was not reached, the 3-year unadjusted survival rates for CAPOX and FOLFOX were 71.6 % (95 % CI, 54.1–83.3) and 68.5 % (95 % CI, 64.2–72.3), respectively (log rank *P* = 0.6737). There were no significant differences in adjusted overall survival between CAPOX and FOLFOX (HR, 1.047; 95 % CI, 0.676–1.622; *P* = 0.8367). The propensity-weighted Cox regression analysis also confirmed these findings (Table 5). The full Cox model included treatment, age, sex, race, positive lymph nodes, tumor grade, comorbidity score, geographic region, and income. After backward elimination, age and tumor grade maintained statistical significance in the model.

**Table 5:** Multivariate Cox regression of overall survival (CAPOX vs. FOLFOX)

Covariates	*N*	Multivariate Cox regression reduced model^a^			Propensity weighted Cox regression^b^		
		HR	95 % CI	*P* value	HR	95 % CI	*P* value
Treatment							
FOLFOX (ref)	1,298	1.000			1.000		
CAPOX	122	1.047	0.676–1.622	0.8367	1.129	0.749–1.699	0.5626
Age at diagnosis							
66–70 (ref)	538	1.000					
71–75	449	1.056	0.795–1.402	0.7071			
76–80	334	1.207	0.892–1.633	0.2217			
>80	99	1.731	1.138–2.633	0.0104			
Tumor grade							
1–3 (ref)	940	1.000					
3–4	437	1.678	1.318–2.137	<0.0001			

^a^Reduced model by backward elimination. Full model included age, sex, race, positive lymph nodes, tumor grade, comorbidity score, geographic region, and income

^b^Propensity score weighted for age, sex, race, positive lymph nodes, tumor grade, comorbidity score, geographic region, and income

## Discussion

Clinical trials confirm that therapy with agents including 5-FU, oxaliplatin, capecitabine, and oxaliplatin is associated with improved survival of patients with stage III and IV mCRC [3–5, 7, 8, 10–16, 18, 36–43]. Importantly, compared with younger patients in the setting of clinical trials, this population-based retrospective cohort analysis of elderly patients in community settings revealed comparable benefits in overall survival and complications requiring medical resource utilization in response to these treatments [44, 45].

The finding that patients treated with CAP were older and had a higher comorbidity burden compared with the three other treatment groups may reflect a belief among physicians that elderly patients are frailer and less able to tolerate aggressive or more toxic treatments. A recent review of the medical records of patients aged 65 or older diagnosed with stage III colon cancer between 2003 and 2006 revealed that 61 % received a regimen containing oxaliplatin, 54 % were treated with FOLFOX, 19 % received 5-FU/LV, and 12 % were administered with capecitabine monotherapy. Among those not treated with oxaliplatin, the primary reason was comorbid health conditions with age cited as a reason for not administering oxaliplatin for 19 % of patients [27].

Patient characteristics such as age, gender, race, and comorbidity burden appear to be important factors in prescribing chemotherapy treatment, but after adjusting for these factors, there were no significant differences in OS between the CAP-based and 5-FU/LV-based regimens. This is an encouraging finding for all patients diagnosed with advanced stage CRC, suggesting that currently available and recommended systemic therapies are equally effective for patients with diverse clinical and demographic characteristics.

Our study observed that complications requiring medical resource utilization were less frequent for CAP ± oxaliplatin regimens while achieving an equivalent survival benefit compared with 5-FU/LV ± oxaliplatin regimens. This confirms similar observations from randomized clinical trials that CAP monotherapy is associated with a lower rate of adverse events and reduced medical resource utilization [8, 46]. Randomized clinical trials report comparative safety profiles for CAP ± oxaliplatin regimens vs. 5-FU/LV ± oxaliplatin with more grade 3 or 4 neutropenia and neutropenic fever associated with FOLFOX and more grade 3 hand–foot syndrome and grade 3 or 4 diarrhea associated with CAPOX [11, 18, 47]. However, the incidence of medically significant diarrhea, i.e., requiring medical resources, was reduced in patients receiving CAPOX vs. FOLFOX in our study. Dose selection was at the discretion of the physician and dosing information could not be determined retrospectively from available data within the claims dataset. Regional differences in tolerance to FP, both CAP and 5-FU/LV, have been reported with US patients more likely to experience grade 3 or 4 FP-related toxicities compared with patients from other parts of world, particularly Asia [48–50]. Physicians may have elected to use doses (lower) and/or treatment schedules other than those tested in clinical trials that may have impacted the safety profile of the regimen. These findings confirm that capecitabine-based treatments can be delivered to elderly patients under the conditions of routine medical care with outcomes similar to those achieved in overall clinical trial populations for patients ≥65 years of age.

Initiation of chemotherapy for all four treatment regimens was longer (mean time, 74 to 81 days) than the typical 30 days that would be expected. Prior research has shown that not only do treatment rates decline dramatically with increasing age [51], but older age is associated with delayed chemotherapy initiation [52] and lower rates of chemotherapy completion [53]. These age disparities in treatment patterns are associated with higher mortality [52, 53] and our results provide further support that demographic factors such as age should not discourage the use of guideline-recommended therapies.

### Study Strengths and Limitations

This study has several strengths, including the large sample size from a population-based registry with a wide geographic representation of patients with CRC in the USA. The SEER–Medicare dataset provides inpatient and outpatient data, comprehensive information about covered services, all claims regardless of residence or care out of area, and longitudinal data with claims for services from the time a person is eligible for Medicare until the date of death. However, use of the SEER–Medicare data for this type of analysis has some limitations, particularly for determining accurate utilization rates of oral chemotherapeutic agents such as capecitabine. A recent comparison of Medicare claims with the National Cancer Institute's Patterns of Care studies showed that among patients with various cancers receiving chemotherapy (including stage II/III CRC), Medicare claims data more accurately identified agents that were intravenously administered [54].

In addition, the SEER–Medicare database does not provide data on performance status or lifestyle factors, such as smoking. These factors could have affected the treatment patterns we observed or clinicians' initial decisions to treat these patients. Furthermore, treatment patterns for the older population with Medicare coverage may be different from those used for younger patients and, therefore, the results might have limited applicability to younger populations in real-world settings. This analysis also does not yield information about patients enrolled in HMOs since these data are not collected by Medicare. It is conceivable that treatment patterns, prognosis, and complications may differ between HMO and Medicare enrollees. Previous studies found that Medicare HMO enrollees with colon cancer had better OS compared with fee-for-service (FFS) plan members [55, 56]. These mortality differences might have been due to higher use of screening and preventive services for HMO patients or the possibility that HMO enrollees tend to be healthier than FFS enrollees.

## Conclusions

Overall survival for elderly mCRC patients who were treated under conditions of routine medical oncology practice was comparable between CAP and 5-FU/LV and between CAPOX and FOLFOX. These results are consistent with those reported among younger patients in randomized clinical trials. The rate of treatment-related complications requiring medical resource use was lower for patients administered capecitabine monotherapy and in combination with oxaliplatin compared with 5-FU/LV and FOLFOX, respectively. These findings confirm that capecitabine-based regimens are an appropriate treatment choice for elderly patients with mCRC. These data also offer support for the use of treatments for elderly patients that are consistent to those administered to younger patients and imply that age should not discourage the use of guideline-recommended therapies for mCRC.

Further research is required to evaluate patterns and outcomes of care for patients with varying performance status since this information is not included in the SEER–Medicare database and we were unable to examine possible interactions between performance status and prognosis and the incidence of treatment-related complications. Another area that warrants further research is a comparison of the treatment patterns, prognosis, and complications of patients enrolled in HMOs compared with those in FFS plans.

## Electronic supplementary material

Below is the link to the electronic supplementary material.

Supplementary Fig. 1 Schematic of inclusion/exclusion process (JPEG 75 kb)

High-resolution image (TIFF 90 kb)
