# Supplementary figures and images for: Comparative Effectiveness of Chemotherapy in Elderly Patients with Metastatic Colorectal Cancer
Source: J Gastrointest Cancer. 2012 Nov 7;44(1):79–88. doi: 10.1007/s12029-012-9450-x (PMC3568483; doi:10.1007/s12029-012-9450-x)

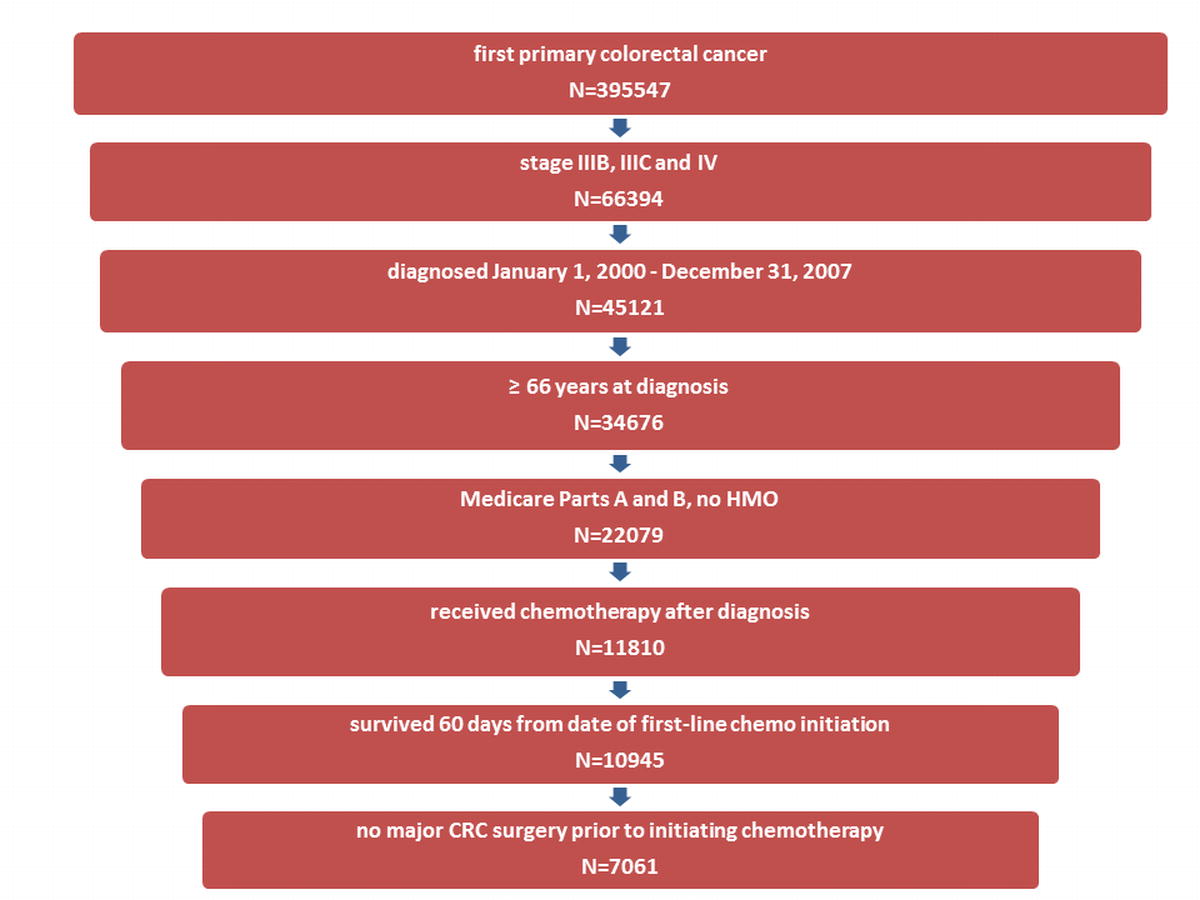

Supplement: Supplementary file 1 — Schematic of inclusion/exclusion process (JPEG 75 kb) [file 12029_2012_9450_Fig4_ESM.jpg]

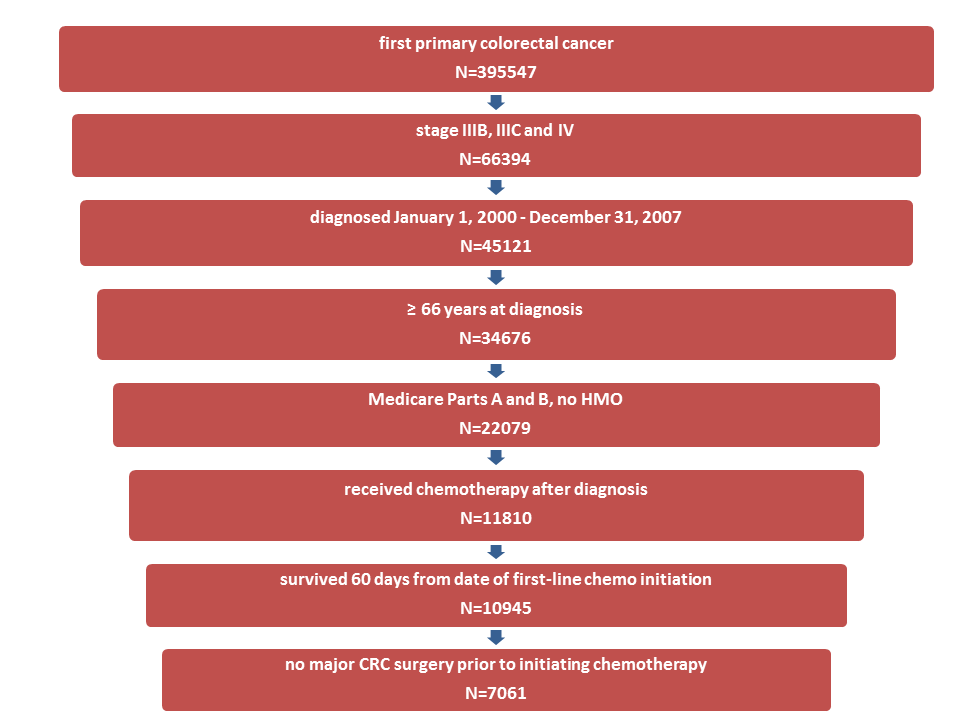

Supplement: Supplementary file 2 — High-resolution image (TIFF 90 kb) [file 12029_2012_9450_MOESM1_ESM.tif]
